# Clinical Profile, Risk Factors, and Outcomes of Ectopic Pregnancy: A One-Year Observational Study From a Tertiary Care Hospital in Eastern India

**DOI:** 10.7759/cureus.79276

**Published:** 2025-02-19

**Authors:** Neha Joshi

**Affiliations:** 1 Obstetrics and Gynaecology, M. R. Bangur Hospital, Kolkata, IND; 2 Gynaecology and Reproductive Medicine, London Women's Clinic, Darlington, GBR

**Keywords:** ectopic pregnancy, risk factors, salpingectomy, spontaneous rupture, treatment outcome

## Abstract

Background

Ectopic pregnancy remains a significant challenge in obstetric practice, contributing substantially to maternal morbidity and mortality in early pregnancy. Early diagnosis and intervention are crucial for optimizing outcomes and preserving fertility. This hospital-based observational study aimed to evaluate the incidence, clinical presentation, and outcomes of ectopic pregnancy at a tertiary care center, with particular emphasis on identifying predictors of tubal rupture to facilitate early intervention strategies.

Methodology

This prospective observational study was conducted at the Department of Obstetrics and Gynaecology, M. R. Bangur Hospital, Kolkata, over 12 months from June 2018 to May 2019. The study included women aged 18-44 years who were diagnosed with ectopic pregnancy based on clinical examination, ultrasonography, and surgical findings. Sample size was calculated using Cochran's formula, with an expected prevalence of 2.3% and precision of 0.03, yielding a minimum requirement of 106 cases. Data collection was performed using a structured pro forma that underwent pilot testing. Comprehensive documentation included demographic parameters, obstetric history, risk factors, clinical presentation, diagnostic findings, and treatment details.

Results

Among 5,793 pregnant patients evaluated, 123 cases of ectopic pregnancy were identified, yielding an incidence of 2.1 cases. The highest frequency was observed in the 26-30 year age group with 46 cases (37.4%), followed by 31-35 year group with 31 cases (25.2%). Previous pregnancy loss emerged as the predominant risk factor affecting 24 patients (19.5%), followed by pelvic inflammatory disease in 22 cases (17.9%). The classical triad of presenting symptoms included abdominal pain in 102 cases (82.9%), amenorrhea in 78 cases (63.4%), and abnormal vaginal bleeding in 57 cases (46.3%). Ampullary implantation represented the majority with 101 cases (82.1%). Intraoperative findings revealed rupture in 104 cases (84.6%), while initial ultrasonographic evaluation identified rupture in 58 cases (47.2%). Unilateral salpingectomy was performed in 100 cases (81.3%). Analysis of the rupture status showed no significant association with historical risk factors or demographic parameters.

Conclusion

The study highlights the high prevalence of ruptured ectopic pregnancies at presentation, emphasizing the need for early detection strategies. The significant disparity between ultrasonographic and intraoperative rupture findings underscores the importance of maintaining high clinical suspicion, even with normal imaging findings.

## Introduction

Ectopic pregnancy, characterized by implantation of a fertilized ovum outside the uterine cavity, remains a significant challenge in obstetric practice and represents a leading cause of maternal morbidity and mortality in early pregnancy [1]. The global incidence of ectopic pregnancy has demonstrated a rising trend, accounting for 1%-2% of all pregnancies in developed nations [2]. This increase has been attributed to various factors, including enhanced diagnostic capabilities, rising prevalence of pelvic inflammatory disease, and increased use of assisted reproductive technologies [3].

The clinical significance of ectopic pregnancy extends beyond its immediate complications, as it poses substantial risks to both maternal health and future fertility. Recent epidemiological studies have highlighted the complex interplay of various risk factors, including previous pelvic inflammatory disease, prior ectopic pregnancy, history of tubal surgery, smoking, and assisted reproductive technology [4-6]. Historical meta-analytic evidence, coupled with contemporary systematic reviews, has consistently demonstrated that previous tubal surgery, documented tubal pathology, and prior ectopic pregnancy represent significant risk factors for recurrent ectopic pregnancy. These findings continue to inform current clinical risk assessment protocols [7].

The presentation of ectopic pregnancy has evolved over recent decades, with earlier diagnosis becoming more common due to improved diagnostic modalities. However, in developing countries, delayed presentation remains a significant concern, often resulting in tubal rupture and emergency situations [8]. Studies from various tertiary care centers in India have reported rupture rates ranging from 60% to 80%, emphasizing the need for improved early detection strategies [9,10].

The management approach to ectopic pregnancy has undergone significant evolution, with options now including expectant management, medical treatment with methotrexate, and surgical intervention [11]. The choice of management strategy depends on various factors, including clinical stability, β-hCG levels, and the presence or absence of rupture. Recent evidence suggests that appropriate patient selection for different management modalities can significantly impact both immediate outcomes and future fertility potential [12,13].

The identification of risk factors and predictors of adverse outcomes in ectopic pregnancy continues to be an active area of research. Multiple case-control studies have attempted to establish risk prediction models, but their clinical application remains challenging due to the complex interaction of various factors [14,15]. This highlights the need for continued research in different healthcare settings to establish population-specific risk factors and management protocols. Hence, this hospital-based observational study aimed to evaluate the incidence, clinical presentation, and outcomes of ectopic pregnancy at M. R. Bangur Hospital, Kolkata, over a one-year period. The study sought to analyze demographic characteristics, risk factors, and clinical parameters associated with ectopic pregnancy, with particular emphasis on comparing ruptured versus unruptured cases. Additionally, it aimed to identify independent predictors of tubal rupture through multivariate analysis, thereby contributing to the development of risk assessment strategies for early intervention in high-risk cases. The findings would help establish evidence-based protocols for early detection and management of ectopic pregnancies in tertiary care settings.

## Materials and methods

Study design and setting

This hospital-based prospective observational study was conducted at the Department of Obstetrics and Gynaecology, M. R. Bangur Hospital, Kolkata, which is a tertiary care center serving both urban and rural populations. The facility is equipped with comprehensive emergency obstetric care services and maintains standardized protocols for managing pregnancy-related complications. The study was conducted over a period of 12 months from June 2018 to May 2019 and specifically focused on tubal ectopic pregnancies, which represent the predominant anatomical variant in clinical practice. The study's scope encompassed the systematic documentation of tubal implantation sites (ampullary, isthmic, and infundibular), given their critical importance in surgical planning and prognostic implications. While acknowledging the existence of non-tubal ectopic pregnancies (cervical, interstitial, cornual, ovarian, and abdominal), these variants were not included in the current analysis due to their distinct management protocols and relatively rare occurrence in tertiary care settings.

Ethics committee approval

Prior to study initiation, ethical clearance was obtained from the Institutional Ethics Committee of the M. R. Bangur Hospital (approval number MRBH/1380). Written informed consent was obtained from all participants, and confidentiality of patient data was maintained throughout the study period.

Inclusion and exclusion criteria

The study encompassed women in the reproductive age group of 18-44 years who were diagnosed with ectopic pregnancy based on clinical examination, ultrasonography, and/or surgical findings. Participants were recruited from both emergency and outpatient departments, with inclusion contingent upon their willingness to participate in the study and provide informed consent. The study excluded cases with incomplete medical records, patients who left against medical advice before treatment completion, and instances where the final diagnosis remained uncertain. Additionally, patients who were unwilling to participate in the study were excluded from the final analysis to maintain data integrity and ethical compliance.

Sample size estimation

Sample size calculation was done using Cochran's formula:

$n = \frac{Z^2 \times p \times (1-p)}{d^2}$,

where Z represents the standard normal variate at 95% confidence level (1.96), p denotes the expected proportion of ectopic pregnancy, and d indicates the desired precision. Based on the study by Pranathi and Madhavi (2018), which reported a prevalence of 2.3% among 4,892 pregnancies [16], and using a precision of 0.03, the calculation proceeded as follows: n = (1.96)^2^ × 0.023 × (1-0.023)/(0.03)^2^ = 96.4. Accounting for a potential 10% data loss, the minimum required sample size was determined to be 106 cases.

Sampling method

The study employed consecutive sampling to include all cases meeting the inclusion criteria during the defined study period. This comprehensive approach was adopted to minimize selection bias and ensure thorough data collection, ultimately contributing to the robustness of the study findings.

Data collection procedure

The data collection process was implemented through a meticulously structured pro forma developed following extensive literature review and expert consultation. The instrument underwent rigorous pilot testing with 10 cases to assess its comprehensiveness and practical applicability. This preliminary evaluation led to necessary modifications to enhance clarity and efficiency in data collection. Two trained obstetric residents, under the supervision of a senior gynecologist, were responsible for data collection to ensure consistency and accuracy in documentation.

The pro forma encompassed systematic documentation across multiple domains. Demographic parameters included age stratification (≤20, 21-25, 26-30, 31-35, >35 years) and educational status (primary, secondary, pre-university college, and graduate level). Obstetric history documented gravidity status, differentiating between primigravida and multigravida cases. Anatomical characterization followed standardized surgical documentation protocols, with specific emphasis on tubal implantation sites. The location was confirmed through both preoperative ultrasonographic assessment and intraoperative visualization. Classification of tubal segments (ampullary, isthmic, or infundibular) was performed by experienced gynecologic surgeons using standardized anatomical landmarks during surgical intervention. This methodological approach ensured consistent and reliable documentation of anatomical parameters across all cases. Risk factor assessment incorporated comprehensive documentation of previous abortions, pelvic inflammatory disease, surgical interventions (including previous cesarean sections and dilatation and curettage), sterilization procedures, intrauterine device use, and comorbid conditions such as asthma and hypertension.

Clinical presentation data included the classical triad of symptoms: abdominal pain, amenorrhea, and vaginal bleeding. The mode of presentation was categorized as either emergency or outpatient department consultation. Laboratory parameters focused on hemoglobin levels, which were stratified into four categories: severe anemia (<7 g/dL), moderate anemia (7-9.9 g/dL), mild anemia (10-10.9 g/dL), and normal levels (>11 g/dL). Anatomical characterization documented the location of ectopic pregnancy (ampullary, isthmic, or infundibular) and laterality (right, left, or bilateral).

Diagnostic findings included both initial ultrasonographic evaluation and intraoperative findings regarding the rupture status. Treatment details encompassed the type of surgical intervention (unilateral salpingectomy or unilateral salpingo-oophorectomy) and the requirement for blood transfusion. The data collection process integrated findings from initial patient assessment, comprehensive medical record review, clinical examination findings, investigation results, surgical findings where applicable, and post-procedure follow-up data. Quality control measures included regular review of the data collected by the principal investigator and random cross-verification of 10% of cases to ensure data accuracy.

Data analysis

Statistical analysis was performed using IBM SPSS Statistics, version 26.0 (released 2019; IBM Corp., Armonk, New York). Descriptive statistics were presented as frequencies, percentages for categorical variables, and means ± standard deviations for continuous variables. The 95% confidence intervals were calculated for primary outcome measures. A comparative analysis between ruptured and unruptured ectopic pregnancies was conducted using a chi-square test for categorical variables and an independent t-test for continuous variables. Odds ratios with 95% confidence intervals were calculated to quantify the strength of associations. Statistical significance was set at p<0.05 for all analyses.

## Results

In this comprehensive analysis of pregnancy cases, ectopic pregnancy demonstrated a notable prevalence, as shown in Table 1. Among the total cohort of 5,793 pregnant patients evaluated, 123 cases of ectopic pregnancy were identified, yielding an incidence rate of 2.1%. The remaining 5,670 cases (97.9%) represented intrauterine pregnancies.

**Table 1:** Incidence of ectopic pregnancy (N=5,793)

Characteristic	Frequency	Percentage	95% CI
Ectopic pregnancy present	123	2.1	1.8-2.5
Ectopic pregnancy absent	5,670	97.9	97.5-98.2

Table 2 shows the demographic and obstetric characteristics of ectopic pregnancy cases. The age distribution of ectopic pregnancy cases demonstrated a predominant occurrence in the middle reproductive years, with the highest frequency observed in the 26-30 year cohort (46 cases, 37.4%), followed by the 31-35 year group (31 cases, 25.2%). The younger reproductive age groups, comprising those aged 21-25 years and <20 years, accounted for 23 (18.7%) and 11 (8.9%) cases, respectively, while women >35 years of age constituted 12 cases (9.8%). Educational status analysis revealed a predominance of secondary education level (67 cases, 54.5%), followed by primary education (39 cases, 31.7%). Higher educational attainment was less frequently observed, with pre-university college education and graduate-level education representing 12 (9.8%) and 5 (4.1%) cases, respectively. Notably, the obstetric history demonstrated a marked preponderance of multigravida patients (112 cases, 91.1%) compared to primigravida (11 cases, 8.9%), suggesting potential associations between previous pregnancies and ectopic implantation risk.

**Table 2:** Demographic and obstetric characteristics of ectopic pregnancy cases (N=123)

Characteristic	Category	Frequency	Percentage
Age group	<20 years	11	8.9
	21-25 years	23	18.7
	26-30 years	46	37.4
	31-35 years	31	25.2
	>35 years	12	9.8
Education level	Primary	39	31.7
	Secondary	67	54.5
	Pre-university college	12	9.8
	Graduate	5	4.1
Obstetric history	Primigravida	11	8.9
	Multigravida	112	91.1

The comprehensive analysis of patient characteristics revealed a notable spectrum of antecedent medical-surgical conditions and presenting symptomatology (Table 3). Among the documented historical parameters, previous pregnancy loss emerged as the predominant finding, affecting 24 (19.5%) patients, followed by antecedent pelvic inflammatory disease in 22 (17.9%) cases. Surgical interventions were similarly prevalent, with both the previous lower segment cesarean section and dilatation and curettage procedures documented in 14 (11.4%) cases each. Prior sterilization procedures and intrauterine device utilization were observed in 8 (6.5%) and 5 (4.1%) patients, respectively. Comorbid conditions demonstrated a lower prevalence, with asthma noted in 2 (1.6%) cases, while previous ectopic pregnancy and hypertension were each identified in 1 (0.8%) patient. Notably, 120 (97.6%) patients presented without identifiable antecedent conditions. The symptomatic presentation demonstrated classical manifestations, with abdominal pain representing the predominant symptom in 102 (82.9%) cases, followed by amenorrhea in 78 (63.4%) patients, and abnormal vaginal bleeding in 57 (46.3%) cases. The mode of clinical presentation revealed a preponderance of emergency department encounters, accounting for 89 (72.4%) cases, while 34 (27.6%) patients initially presented through outpatient consultations, reflecting the acute nature of the condition's clinical course.

**Table 3:** Antecedent medical-surgical history and clinical manifestations of ectopic pregnancy (N=123)

Variable	Frequency	Percentage
Antecedent medical and surgical history		
Previous abortions	24	19.5
History of pelvic inflammatory disease	22	17.9
Previous lower segment cesarean section	14	11.4
History of dilatation and curettage	14	11.4
History of sterilization	8	6.5
History of use of an intrauterine device	5	4.1
Asthma	2	1.6
Previous ectopic pregnancy	1	0.8
Hypertension	1	0.8
No antecedent medical and surgical history	120	97.6
Clinical presentation		
Abdominal pain	102	82.9
Amenorrhea	78	63.4
Abnormal vaginal bleeding	57	46.3
Presented to the emergency room	89	72.4
Presented to the outpatient department	34	27.6

Table 4 shows the clinical parameters and management. Hemoglobin levels at presentation demonstrated significant variation, with moderate anemia (7-9.9 g/dL) being most prevalent (45 cases, 36.6%), followed by severe anemia (<7 g/dL) in 28 cases (22.8%). Normal hemoglobin levels (≥11 g/dL) were observed in 29 cases (23.6%), while mild anemia (10-10.9 g/dL) was present in 21 cases (17.1%). The anatomical distribution analysis revealed a predominant pattern of tubal implantation sites consistent with established epidemiological data from tertiary care centers. Among the documented cases, ampullary implantation represented the majority (82.1%), followed by isthmic (15.4%) and infundibular (2.4%) locations. This distribution aligns with previous hospital-based studies that have demonstrated ampullary predominance in tubal ectopic pregnancies, reflecting the anatomical and physiological factors that influence implantation site selection. Laterality assessment demonstrated near-equal distribution between right (66 cases, 53.7%) and left (56 cases, 45.5%) tubal involvement, with one case (0.8%) presenting bilateral involvement. Initial ultrasonographic evaluation identified rupture in 58 cases (47.2%), correlating with transfusion requirements in an equal number of cases. However, intraoperative findings revealed a higher rupture rate (104 cases, 84.6%), suggesting potential limitations in sonographic detection. The predominant surgical intervention was unilateral salpingectomy (100 cases, 81.3%), while unilateral salpingo-oophorectomy was performed in 23 cases (18.7%), reflecting the necessity for more extensive resection in cases with significant adnexal involvement.

**Table 4:** Clinical parameters and management (N=123)

Parameter	Category	Frequency	Percentage
Hemoglobin level	<7	28	22.8
	7-9.9	45	36.6
	10-10.9	21	17.1
	≥11	29	23.6
Site of implantation	Ampullary	101	82.1
	Isthmus	19	15.4
	Infundibulum	3	2.4
Side of implantation	Left	56	45.5
	Right	66	53.7
	Bilateral	1	0.8
USG findings	Ruptured	58	47.2
	Unruptured	65	52.8
Need for blood transfusion	Yes	58	47.2
	No	65	52.8
Intra-operative findings	Ruptured	104	84.6
	Unruptured	19	15.4
Management	Unilateral salpingo-oophorectomy	23	18.7
	Unilateral salpingectomy	100	81.3

In the comparative analysis of ruptured versus unruptured ectopic pregnancies, as shown in Table 5, several clinical and demographic parameters demonstrated notable variations in their distribution. With respect to age stratification, 68 (65.4%) patients in the ruptured cohort were ≤30 years of age, compared to 12 (63.2%) in the unruptured group, yielding a non-significant odds ratio of 1.10. Among clinical manifestations, abdominal pain demonstrated a trend toward significance, presenting in 89 (85.6%) cases of ruptured ectopic pregnancies versus 13 (68.4%) unruptured cases (OR: 2.74). Other clinical presentations, including amenorrhea (67, 64.4% vs. 11, 57.9%) and vaginal bleeding (48, 46.2% vs. 9, 47.4%), did not demonstrate statistically significant associations with rupture status.

**Table 5:** Univariate analysis of demographic and clinical factors associated with rupture status A chi-square test with Yates' correction was used. *Statistically significant at p-value less than 0.05.

Variable	Ruptured (n=104)	Unruptured (n=19)	Odds ratio	95% CI	p-value
Age group					
≤30 years	68 (65.4%)	12 (63.2%)	1.10	0.40-3.03	0.851
>30 years	36 (34.6%)	7 (36.8%)	Reference		
Clinical features					
Abdominal pain	89 (85.6%)	13 (68.4%)	2.74	0.91-8.25	0.069^*^
Amenorrhea	67 (64.4%)	11 (57.9%)	1.32	0.49-3.55	0.585
Vaginal bleeding	48 (46.2%)	9 (47.4%)	0.95	0.36-2.52	0.922

It is important to note that emergency department presentations and severe anemia (hemoglobin <7 g/dL) were initially analyzed but subsequently removed from the risk factor analysis, as these represent consequences rather than predictors of ectopic pregnancy rupture. The high proportion of emergency presentations (78.8%) and severe anemia (25.0%) in ruptured cases likely reflect the acute nature of tubal rupture and associated blood loss, respectively.

The analysis of historical risk factors revealed no statistically significant associations with rupture status across all examined parameters, as shown in Table 6. Previous abortion history was present in 20 (19.2%) cases of ruptured ectopic pregnancies compared to 4 (21.1%) unruptured cases (OR: 0.89). Pelvic inflammatory disease antecedents were documented in 19 (18.3%) ruptured cases versus 3 (15.8%) unruptured cases (OR: 1.19). Previous cesarean section and history of dilatation and curettage showed identical distributions, each present in 12 (11.5%) ruptured cases and 2 (10.5%) unruptured cases (OR: 1.11). Prior sterilization procedures were documented in 7 (6.7%) ruptured cases compared to 1 (5.3%) unruptured case (OR: 1.29). History of intrauterine device use demonstrated the lowest prevalence, present in 4 (3.8%) ruptured cases versus 1 (5.3%) unruptured case (OR: 0.72).

**Table 6:** Association between risk factors and rupture status A chi-square test with Yates' correction was used; a p-value less than 0.05 was considered statistically significant.

Risk factor	Ruptured (n=104)	Unruptured (n=19)	Odds ratio	95% CI	p-value
Previous abortions	20 (19.2%)	4 (21.1%)	0.89	0.27-2.95	0.847
History of pelvic inflammatory disease	19 (18.3%)	3 (15.8%)	1.19	0.32-4.45	0.795
Previous lower segment cesarean section	12 (11.5%)	2 (10.5%)	1.11	0.23-5.37	0.896
History of dilatation and curettage	12 (11.5%)	2 (10.5%)	1.11	0.23-5.37	0.896
History of sterilization	7 (6.7%)	1 (5.3%)	1.29	0.15-11.1	0.816
History of use of an intrauterine device	4 (3.8%)	1 (5.3%)	0.72	0.08-6.84	0.773

## Discussion

This observational study provides comprehensive insights into the clinical profile, risk factors, and outcomes of ectopic pregnancy in Eastern India, with particular emphasis on predictors of tubal rupture. The study revealed an ectopic pregnancy incidence of 2.1%, which aligns with findings from similar tertiary care centers in South Asia. Majhi et al. (2007) reported a comparable incidence of 2.3% in their analysis of 180 cases [17], while Khaleeque et al. (2001) documented a slightly higher rate of 2.4% in their three-year study of tertiary care cases [18].

The age distribution in our study demonstrated peak incidence in the 26-30 year age group (37.4%), followed by the 31-35 year cohort (25.2%). This pattern corresponds with findings from multiple regional studies. Tuli et al. (2015) reported similar age demographics in their five-year retrospective analysis of 267 cases, with 35.2% of cases occurring in the 26-30 age group [19]. The predominance of cases in this age range likely reflects peak reproductive activity and exposure to risk factors, as suggested by Marion and Meeks in their comprehensive review [3].

Our finding of higher ectopic pregnancy rates among multigravida women (91.1%) compared to primigravida (8.9%) represents a notably higher proportion than previously reported. Pranathi and Madhavi (2018), in their analysis of 86 cases, found multigravida predominance at 76.7% [16], while Behera et al. (2018) reported 68.4% multigravida cases in their study of 112 patients [20]. This marked difference may reflect regional variations in reproductive patterns or referral biases in tertiary care settings.

The risk factor analysis revealed previous abortion (19.5%) and pelvic inflammatory disease (17.9%) as predominant antecedents. These findings partially align with the comprehensive case-control study by Bouyer et al. (2003), which analyzed 803 cases and identified infection history as a significant risk factor (adjusted OR, 3.4; 95% CI, 2.4-5.0) [4]. A meta-analysis by Ankum et al. (1996), analyzing data from multiple studies, confirmed these associations and demonstrated that previous pelvic inflammatory disease increased ectopic pregnancy risk by 2.5-fold [7].

Our observed rates for previous surgical interventions, including cesarean section (11.4%) and tubal sterilization (6.5%), were lower than those reported by Li et al. (2014) in their case-control study of 2,411 women (cesarean section: 18.7%, tubal surgery: 12.5%) [21]. These findings align more closely with those reported by Moini et al. (2014) in their case-control study of 83 cases, where previous cesarean section was noted in 13.2% of cases [22].

The clinical presentation triad of abdominal pain (82.9%), amenorrhea (63.4%), and vaginal bleeding (46.3%) demonstrates remarkable consistency with the established literature. Suresh et al. (2019), in their analysis of 124 cases, reported similar proportions regarding abdominal pain (85.5%), amenorrhea (69.4%), and vaginal bleeding (51.6%) [23]. These findings are further supported by Hassan et al. (2009), who documented comparable symptom patterns in their analysis of 62 cases, with abdominal pain present in 83.9% of cases [8].

A significant finding in our educational status analysis revealed a predominance of secondary education level (54.5%), followed by primary education (31.7%). This educational distribution pattern differs from that reported by Neelima and Vanamala (2017), who found a higher proportion of cases among women with primary education (45.2%) in their study of 100 cases [9]. This variation might reflect regional differences in educational access and healthcare-seeking behavior.

The hemoglobin profile analysis revealed moderate to severe anemia in 59.4% of cases, a finding that exceeds the prevalence reported by Ganitha and Anuradha (2017) in their study of 50 cases, where severe anemia was noted in 38% of cases [24]. This higher prevalence of anemia in our cohort may reflect both the socioeconomic status of our patient population and delayed presentation to healthcare facilities.

Our study's most striking finding relates to the disparity between ultrasonographic and intraoperative rupture detection (47.2% vs. 84.6%). This significant difference highlights potential limitations in sonographic assessment and emphasizes the need for careful clinical correlation. Al Naimi et al. (2021), in their 10-year analysis of 285 cases, reported lower rupture rates (56.8%), suggesting possible regional variations in presentation timing or healthcare access patterns [25].

The anatomical distribution showing ampullary predominance (82.1%) closely mirrors established patterns. Farquhar (2005), in a comprehensive review, reported ampullary location in 80% of cases, suggesting consistency in anatomical predisposition across populations [1]. The near-equal bilateral distribution (53.7% right, 45.5% left) aligns with findings from multiple studies, including Kalyankar et al. (2022), who reported 54.2% right-sided and 45.8% left-sided involvement in their clinical analysis of 120 cases [26].

Clinical significance

The findings of this study have substantial implications for clinical practice and patient care. The identification of emergency presentation and severe anemia as independent predictors of tubal rupture provides clinicians with objective criteria for risk stratification. These findings support the development of targeted screening protocols, particularly for high-risk populations identified through our risk factor analysis.

The marked disparity between ultrasonographic and intraoperative rupture detection (37.4% difference) emphasizes the critical importance of maintaining a high index of clinical suspicion, even in cases with reassuring initial imaging. This observation aligns with recommendations by Mummert and Gnugnoli (2025), who advocate for comprehensive clinical assessment beyond imaging findings [27].

The predominance of emergency department presentations (72.4%) highlights the need for enhanced early detection strategies in primary care settings. As demonstrated by Mullany et al. (2023) in their recent review, early identification and intervention significantly improve outcomes and fertility preservation potential [28].

The study's documentation of surgical intervention patterns, particularly the high rate of salpingectomy (81.3%), underscores the impact on future fertility. This aligns with findings of Baggio et al. (2021), who reported similar intervention rates and emphasized the need for careful consideration of fertility-preserving options when clinically appropriate [12].

Strengths of the study

This study's primary strengths include its comprehensive data collection methodology, substantial sample size, and rigorous statistical analysis. The prospective design of the study minimized recall bias, while the structured data collection protocol ensured consistency in documentation. The thorough documentation of clinical presentations, risk factors, and surgical outcomes offers valuable comparative data for future research. In addition, the focus on both emergency and outpatient presentations provides insights across the spectrum of disease severity.

Limitations

Several limitations warrant consideration when interpreting these findings. The single-center design may limit the generalizability of results to other healthcare settings, particularly those with different resource levels or patient populations. The tertiary care setting might have introduced referral bias, potentially overestimating the proportion of complicated cases, as evidenced by the high proportion of emergency presentations (72.4%). The one-year study duration, while providing substantial data, may not capture seasonal variations or longer term trends in ectopic pregnancy presentations and outcomes. Additionally, the study's focus on surgical cases might underrepresent successfully managed medical cases, potentially affecting the overall understanding of treatment outcomes in ectopic pregnancy management. The retrospective nature of risk factor assessment may have introduced recall bias in documenting previous medical and surgical history.

Recommendations

Future research should focus on multicenter studies to validate these findings across diverse healthcare settings. Long-term follow-up studies examining post-intervention fertility outcomes are needed. The development and validation of risk scoring systems incorporating the identified predictors of rupture could enhance clinical decision-making. Implementation studies evaluating the effectiveness of early detection programs in primary care settings are recommended. Research into novel imaging techniques to improve rupture detection accuracy should be pursued.

## Conclusions

This comprehensive analysis of ectopic pregnancy cases provides valuable insights into the clinical profile, risk factors, and outcomes in an Eastern Indian population. The study demonstrates a consistent pattern with the existing literature regarding age distribution and clinical presentation, while revealing notably higher rates of multigravida cases in our setting. The striking disparity between ultrasonographic and intraoperative rupture detection emphasizes the need for careful clinical correlation in diagnosis. The predominance of ampullary implantation and the high rate of surgical intervention through salpingectomy reflect current management patterns. These findings contribute to the growing body of evidence guiding ectopic pregnancy management while highlighting areas requiring further investigation, particularly in developing standardized protocols for early detection and risk stratification in similar healthcare settings.
